# Phylogenomic Evidence of Reinfection and Persistence of SARS-CoV-2: First Report from Colombia

**DOI:** 10.3390/vaccines9030282

**Published:** 2021-03-19

**Authors:** Juan David Ramírez, Marina Muñoz, Nathalia Ballesteros, Luz H. Patiño, Sergio Castañeda, Carlos A. Rincón, Claudia Mendez, Carolina Oliveros, Julie Perez, Elizabeth K. Márquez, Frank de los Santos Ortiz, Camilo A. Correa-Cárdenas, Maria Clara Duque, Alberto Paniz-Mondolfi

**Affiliations:** 1 Centro de Investigaciones en Microbiología y Biotecnología-UR (CIMBIUR), Facultad de Ciencias Naturales, Universidad del Rosario, 110111 Bogotá, Colombia; claudia.munoz@urosario.edu.co (M.M.); nathalia.ballesteros@urosario.edu.co (N.B.); lhpatinoblan@yahoo.com (L.H.P.); scastanedagarzon@gmail.com (S.C.); 2 Grupo de Investigación en Enfermedades Tropicales del Ejército (GINETEJ), Laboratorio de Referencia e Investigación, Dirección de Sanidad Ejército, 110111 Bogotá, Colombia; rinconarangocarlos@gmail.com (C.A.R.); Claudiamendez11@gmail.com (C.M.); Carolinaesm6012@hotmail.com (C.O.); Julieperez8228@gmail.com (J.P.); Marqueze_llk11@hotmail.com (E.K.M.); frankortizserrano@gmail.com (F.d.l.S.O.); camilocc510@gmail.com (C.A.C.-C.); Duquemacla@outlook.com (M.C.D.); 3 Icahn School of Medicine at Mount Sinai, New York, NY 10001, USA; Alberto.Paniz-mondolfi@mountsinai.org; 4 Instituto de Investigaciones Biomédicas IDB/Incubadora Venezolana de la Ciencia, 3001 Barquisimeto, Venezuela

**Keywords:** SARS-CoV-2, reinfections, genomic surveillance, Colombia

## Abstract

The continuing evolution of SARS-CoV-2 and the emergence of novel variants have raised concerns about possible reinfection events and potential changes in the coronavirus disease 2019 (COVID-19) transmission dynamics. Utilizing Oxford Nanopore technologies, we sequenced paired samples of three patients with positive RT-PCR results in a 1–2-month window period, and subsequent phylogenetics and genetic polymorphism analysis of these genomes was performed. Herein, we report, for the first time, genomic evidence of one case of reinfection in Colombia, exhibiting different SARS-CoV-2 lineage classifications between samples (B.1 and B.1.1.269). Furthermore, we report two cases of possible viral persistence, highlighting the importance of deepening our understanding on the evolutionary intra-host traits of this virus throughout different timeframes of disease progression. These results emphasize the relevance of genomic surveillance as a tool for understanding SARS-CoV-2 infection dynamics, and how this may translate effectively to future control and mitigations efforts, such as the national vaccination program.

## 1. Introduction

The coronavirus disease 2019 (COVID-19) has spread, infecting over 115 million people worldwide and approaching 2.6 million deaths globally by March 4, 2021. Despite the arrival of several candidate vaccines, which have effectively started to tackle the ongoing pandemic, several questions remain unanswered regarding the dynamics of COVID-19 infection and most importantly the continuing evolution and ever-changing mutational landscape of SARS-CoV-2 genomes [1]. In recent months, a sustained number of mutations of the viral genome have led to the emergence of “Variants of epidemiological concern”. Amongst these are the British variant (B.1.1.7), Brazilian variant (P.1.) and South African variant (B.1.1.32), the latter two with a key repertoire of mutations in the spike gene linked to the potential occurrence of reinfections [2,3,4].

Reinfections are rare in COVID-19, and few studies have documented such events. Reports from Korea, the Netherlands, the United States of America and Ecuador have established diverse criteria for diagnosing reinfections. However, to date, there is currently no standard case definition criteria for identifying these cases, in part due to the diverse testing approaches employed for diagnosis. Recently, the CDC in an effort to better delineate these cases has proposed a scheme combining investigative criteria for identifying highly suspicious cases along with the genomic testing of paired specimens meeting the minimum quality standards [5]. The identification of paired specimens with phylogenomic proof of distinct lineages remains the mainstay of evidence for SARS-CoV-2 reinfection [6,7,8].

Colombia is among the most highly impacted countries by COVID-19 in South America, with more than 2 million infected people and around 60,000 deaths reported to date. Genomic surveillance deployed by the National Institute of Health suggests the circulation of at least 50 SARS-CoV-2 lineages across the country, including the Brazilian variant (P.1), which poses a proved risk for reinfection [9]. To date, only one study has documented a suspected case of reinfection in Colombia, based solely on epidemiological data [10]. Yet, no studies have further confirmed reinfection events using a whole-genome sequencing approach. Herein, we provide the first genomic evidence of a reinfection case attributed to different SARS-CoV-2 lineages as well as two cases of possible viral persistence in two patients found to be infected by the same lineage over a 1-month interval window. The epidemiological implications of these cases are discussed.

## 2. Materials and Methods

**Figure 1:**
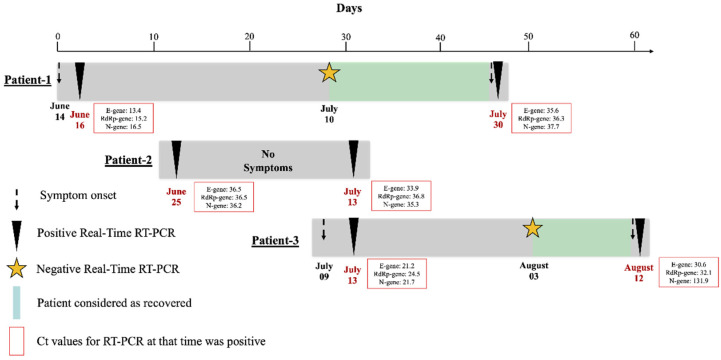
Epidemiological investigation in the three patients. Gray color indicates positive RT-PCR; green indicates a negative PCR. The epidemiological investigation was conducted in 2020.

An epidemiological investigation was conducted in three patients with positive RT-PCR results after being diagnosed with a positive RT-PCR 1 or 2 months prior. Patients were admitted to the Military Hospital of Bogotá, Colombia. The timeline of epidemiological investigations is depicted in Figure 1. This study was performed following the Declaration of Helsinki and its later amendments. The data of the patients were anonymized, which does not represent any risk. The patients provided oral informed consent.

Nasopharyngeal swabs received in the laboratory from all three patients (six paired samples in total) were subjected to automated RNA extraction in an EX3600 Liferiver^®^ platform using the Viral RNA Isolation Kit, (Liferiver Bio-Tech, Shanggai, China) following the manufacturer’s instructions. The addition of an exogenous internal control composed of MS2 phage genome (RP-IC), provided by the diagnostic kit assay, was concurrent with sample preparation. RNA was stored at 4 °C until further processing within 24 h. Molecular detection of SARS-CoV-2 in clinical specimens was performed using the Allplex™ 2019-nCoV assay (Seegene, Korea) as per the kit insert, with unchanged cycling parameters in a CFX96™ Real-Time PCR detection system (Bio-Rad Laboratories, USA). Real-time data analysis was performed using the Seegene 2019-nCoV Viewer Software version 3.18, and target genes included E, RdRP and N, in addition to the RP-IC control detection.

Positive paired samples from patients 1, 2 and 3 were submitted for whole-genome sequencing. Sequence libraries were prepared from RNA extracted from each nasopharyngeal swab per individual using the ARTIC Network protocol (https://artic.network/ncov-2019 accessed on 1 February 2021). Long-read Oxford Nanopore MinION sequencing was conducted by the MinKNOW application (v1.5.5). The raw Fast5 files were base called and demultiplexed using Guppy; then the reads were filtered, eliminating the possible chimeric reads; finally, the genome assemblies were obtained following the MinION pipeline described in the ARTIC bioinformatics pipeline (https://artic.network/ncov-2019/ncov2019-bioinformatics-sop.html accessed on 1 February 2021). Each assembly was typed based on the PANGOLIN nomenclature lineage assigner [11].

Phylogenetic relationships of the obtained genome assemblies were evaluated with a comparative approach including a dataset of 4197 sequences publicly available from the Global Initiative on Sharing All Influenza Data (GISAID) database [12]. The set of assemblies was aligned; the genome SNPs without UTRs were extracted and then were used to build a maximum likelihood phylogeny according with a previously reported methodology [13]. Briefly, a maximum likelihood (ML) tree from the trimmed alignment for the complete dataset was inferred using IQ-TREE 2 [14], considering the best substitution model identified by the default heuristic search option, and ultrafast bootstrapping with 1000 replicates and other parameters by default. Finally, the polymorphisms between the pairs of genomes per patient were evaluated comparing each pair of genomes with each other and with the Wuhan reference sequence (NC_045512), using the UGENE v.33.0 software [15].

## 3. Results

Patient 1 was a one-year-old female with symptoms onset on June 14, who presented mainly with cough and fever. The physician suspected COVID-19 infection and requested an RT-PCR test that was positive on June 16 (Ct = 13.4 E-gene; Ct = 15.2 RdRp-gene; Ct = 16.5 N-gene). A follow-up test was performed on July 10, and the result was negative. The patient returned for a consultation for a new onset of cough on July 30, and RT-PCR at that time was positive (Ct = 35.6 E-gene; Ct = 36.3 RdRp-gene; Ct = 37.7 N-gene). No close positive COVID-19 contacts were reported. The mother of the patient did not report any additional diseases related to the patient or that any members of the family had tested positive for COVID-19.

Patient 2 was a 47-year-old asymptomatic male with a positive RT-PCR test on June 25 (Ct = 36.5 E-gene; Ct = 36.5 RdRp-gene; Ct = 36.2 N-gene). The follow-up test was not reported, and on July 13, the patient returned for a routine follow-up consultation. At that time, the physician requested a repeat RT-PCR test that was positive (Ct = 33.9 E-gene; Ct = 36.8 RdRp-gene; Ct = 35.3 N-gene) with no apparent respiratory symptoms. The patient reported that one member of his family had previously tested positive for COVID-19. The patient did not report any chronic illness.

Patient 3 was a 54-year-old female with symptoms onset on July 9, presenting with cough, fever, odynophagia and fatigue. RT-PCR for SARS-CoV-2 was positive on July 13 (Ct = 21.2 E-gene; Ct = 24.5 RdRp-gene; Ct = 21.7 N-gene). A follow-up test performed on August 3 was negative. On August 12, the patient presented again with recurrent fever and odynophagia and a repeat RT-PCR came back positive (Ct = 30.6 E-gene; Ct = 32.1 RdRp-gene; Ct = 31.9 N-gene). No close positive COVID-19 contacts were reported. The patient presented hypertension, gastritis, and arthrosis; in the clinical history, the use of Losartan, acetylsalicylic acid (ASA) and Omeprazole as treatment was also reported.

All six samples (two positive RT-PCR for SARS-CoV-2 per patient) were submitted for whole-genome sequencing. Genome assemblies with more than 120X coverage depth and more than 97.6% of coverage of the reference genome were obtained for all analyzed samples, with the following paired profiles per patient: (i) Patient 1 with 412.8 coverage depth for the first sample vs. 305.8 coverage depth for the second sample; (ii) Patient 2 with 270.0 coverage depth for the first sample vs. 306.2 coverage depth for the second sample; and (iii) Patient 3 with 438.1 coverage depth for the first sample vs. 122.3 coverage depth for the second sample. The sequences were deposited on GISAID EpiCoV under the numbers EPI_ISL_1040921, EPI_ISL_1040922, EPI_ISL_1040923, EPI_ISL_1040924, EPI_ISL_1040925 and EPI_ISL_1040926.

**Figure 2:**
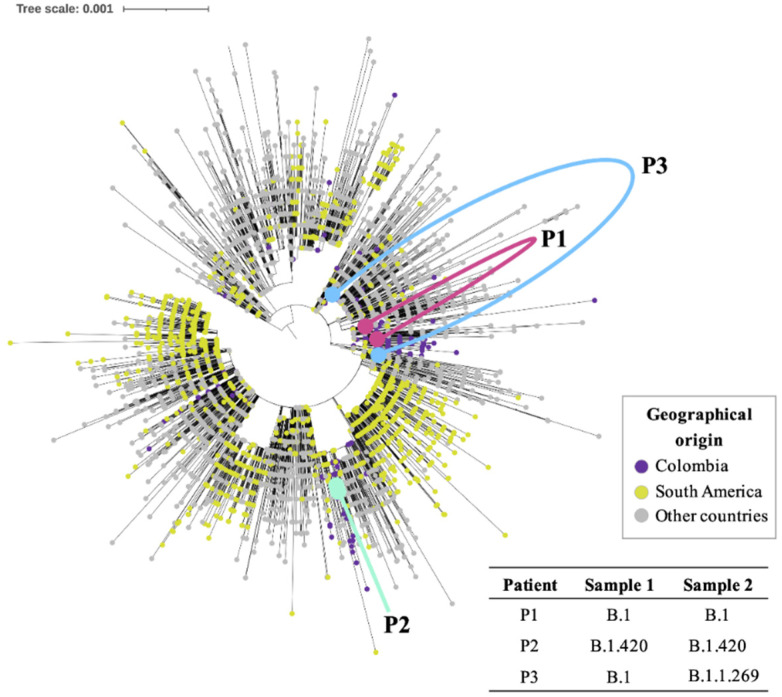
Phylogenomic reconstruction of the six genomes sequenced in this study compared to the 4171 genomes encompassing the global diversity of SARS-CoV2. The red dots and lines represent Patient 1 (persistence); the turquoise dots and lines represent Patient 2 (persistence); and the blue dots and lines represent Patient 3 (reinfection).

**Figure 3:**
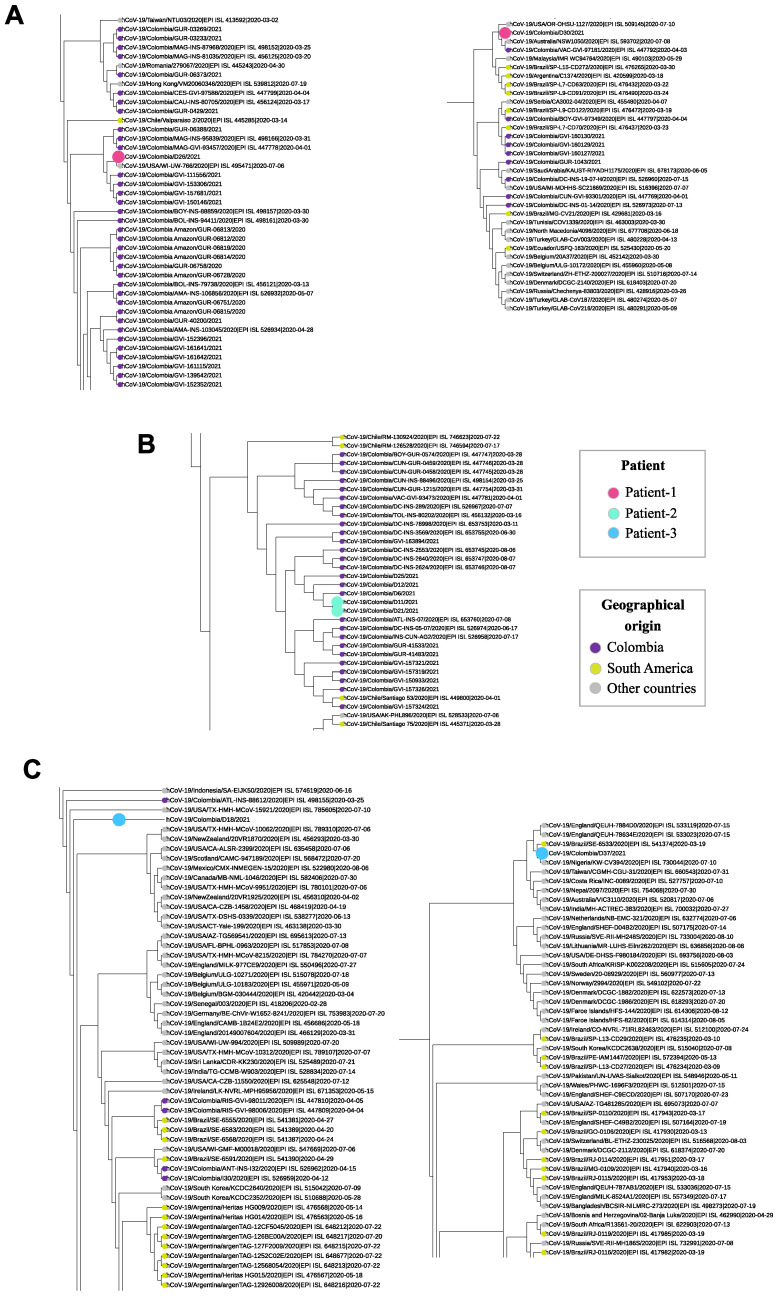
Phylogenetic relationships for the paired genome assemblies obtained from each patient. A zoom to which each of the analyzed genomes belongs was carried out to identify the geographical origin of the most closely related genomes: (**A**) Patient 1; (**B**) Patient 2; (**C**) Patient 3.

The typing and evaluation of the phylogenetic relationships of the analysed genomes, in the context of a dataset with representative genomes of SARS-CoV-2 circulating worldwide (Figure 2; Table S1), displayed that two of the patients had the same lineage in both analysed samples. Two genomes obtained from Patient 1 classified as B.1 lineage, the most abundant and diverse lineage both worldwide and in Colombia [16]. The first genome sequenced from this patient was found to be mostly related to genomes from Colombia; however, its closest genome was one from the United States of America, the only one with a different origin within the cluster of nine genomes which it was part of, while the second genome from Patient 1 was included in a cluster of 32 genomes, which included other nine genomes from Colombia (26.5%), eight genomes from other South American countries (23.5%) and the remaining 16 from other countries (Figure 3A). Two genomes from Patient 2 were assigned to B.1.420 lineage and were closely related within a cluster including 32 genomes mostly from Colombia (n = 29; 90.7%), which also included three genomes from different origins (9.3%), all reported from Chile (Figure 3B). The findings in Patients 1 and 2 support a likely scenario of viral persistence carrying SARS-CoV-2 belonging to the same lineage at the two evaluation times.

In Contrast, Patient 3 was initially found to be infected by lineage B.1 with subsequent characterization of lineage B.1.1.269 in the second sample, suggesting a potential case of reinfection. The first sample of Patient 3 (lineage B.1) was included in a heterogeneous cluster along with 318 other genomes mostly related to genomes from the United States of America (Figure 3C). In this cluster, 45 genomes were identified from Colombia (14.1%), another 34 from South America (10.7%) and the remaining 240 (75.2%) from other countries. The second genome obtained from Patient 3 (lineage B.1.1.269) was closely related to genomes with origins different to Colombia (Figure 3C), and most of them were closely related to genomes from Nigeria and Brazil (Figure 3C)**.**

We further identified variations between them. The paired samples of Patient 1 revealed four substitutions located in ORF1ab (G12160A), S (C21621T) and ORF8 (C28005T and C28093T) genes. One substitution, located in the ORF1ab gene (C5055G), was identified in Patient 2. Finally, the paired samples of Patient 3 exhibited six substitutions in total, two of them located in the ORF1ab gene (C1059T and G3483A), one in the S gene (T23443C) and the remaining three located in the N gene (G28881A, G28882A and G28883C). The coverage of those substitutions sites is graphically represented in Figure S1.

## 4. Discussion

COVID-19 reinfections appear to be rare with very few cases confirmed by whole-genome sequencing reported globally to date [17]. Recently, the emergence of SARS-CoV-2 variants, such as the P.1 and the B.1.351, harboring a mutational repertoire within the Spike gene has raised concerns with regard to how this genetic variability may shape and modulate tissue tropism and potentially preclude the binding of neutralizing antibodies leading to a potential risk for reinfections, as recently reported [16,17,18,19]. Additionally, in a recent report, the immune plasma of COVID-19 convalescent blood donors had 6-fold less neutralizing capacity against the P.1 than against the B-lineage. Moreover, five months after booster immunization with CoronaVac, the plasma from vaccinated individuals failed to efficiently neutralize P.1 lineage isolates [20]. This reinforces the hypothesis of potential emerging reinfections, particularly in South America.

Hence, along with current genomic surveillance efforts directed to tracing emerging variants worldwide, active surveillance for reinfections also becomes a pressing need to effectively control the ongoing pandemic. Continued diversification leading to an increased number of mutations in the S region suggests that reinfection events may be more common than previously thought, even in patients with impaired humoral immunity among which there is a latent risk of reinfections, as suggested in a recent report that revealed a broad spectrum of infectivity, host immune responses and accumulation of mutations, some with the potential for immune escape [21].

Herein, and to the best of our knowledge, we report the first confirmed case of reinfection in Colombia using a whole genome sequencing approach. This case (Patient 3) showed consecutive infections with independent SARS-CoV-2 lineages (Figure 2 and Figure 3), B.1 and later B.1.1.269, two of which are phylogenetically distant while also displaying six substitutions of difference within a two-month window period. The B.1 lineage is highly frequent in Colombia (around 40% of all Colombian SARS-CoV-2 genomes reported to date). This corresponds to the first report of B.1.1.269 in the country, suggesting that the novel detection of lineages explains the mutation of SARS-CoV-2. On the other hand, this case also highlights the need to continue to strengthen efforts towards improving genomic surveillance and expanding sequencing capacity in the country, as the current available number of genomes is scarce (less than 1% of positive COVID-19 cases have been sequenced). Our patient fully recovered after the second infection (no symptoms of chronic COVID-19 illness); yet, this case underscores the vulnerabilities of the host immune response when challenged by variants even between lineages descending from the same major lineage (B as in this case). The B lineage is the most prevalent major lineage circulating in South America [16]. Understanding this variability and the evolutionary dynamics of these emerging variants is pivotal for designing prevention strategies, such as vaccination campaigns, particularly in those countries where vaccination coverage is scarce, thus potentially setting the stage for other reinfection events.

Intriguingly, herein, we also report on two patients with persistent SARS-CoV-2 shedding and low viral loads (Ct values over 30) over an extended time period. Patient 1 recovered from symptoms a month earlier and later presented with recurrent cough. Analyses of the second paired sample revealed four substitutions, strongly suggesting the possibility of intra-host mutation. Nevertheless, we were not able to continue monitoring the viral load of this patient to sequence additional samples, which represented a limitation in our study. A recent study followed 33 patients that tested positive for SARS-CoV-2 for 16 days using whole genome sequencing. The authors highlight that the analysis of viral sequences confirmed persistent infection with evidence for a transmission cluster in health care professionals that shared the same workplace. In this case, the host’s innate immunity shapes the increase in intra-host diversity [22]. In our case, and in light of recent evidence, future studies are needed to fully prove this is a case of intra-host mutation.

Another likely scenario for this case could also be that of reinfection by the same lineage; yet, future investigations are required to fulfill this hypothesis in the absence of molecular clocks on SARS-CoV-2 and frequent convergent evolution [23]. Conversely, for Patient 2, who was initially asymptomatic, phylogenetic analysis revealed the persistence of B.1 lineage with only one substitution between the paired samples. Persistence has been widely documented in COVID-19, characterized by extensive lung thrombosis, long-term persistence of viral RNA in pneumocytes and endothelial cells, along with the presence of infected cell syncytia [24]. This feature has been mainly reported in patients with impaired immunity or cancer around the globe [25,26]. However, information of SARS-CoV-2 persistence using whole-genome sequencing is scarce, with only one case of follow-up in an immunocompromised patient [27].

Positive selection stands as one of the most likely scenarios promoting virus adaptation and lineage-specific selection by attempting to relieve the pressure imposed by the host [20], thus setting the stage for possible cases of reinfection. On the other hand, prolonged infection in an immunocompromised host may contribute to relieving the virus from the pressure exerted by the immune system, leading to insidious intra-host mutation events and adaptation, which may also contribute to the emergence of new virus variants. Further surveillance and follow-up studies are needed to clearly understand the evolutionary pathways leading to SARS-CoV-2 infection and persistence, particularly in the same individuals and at different timeframes.

Our sequencing strategy employed a widely used protocol pipeline for SARS-CoV-2. The coverage and depth obtained from the six genomes presented herein are consistent with high-quality genomes previously reported for SARS-Co-V2 [28]. However, it is well documented that Oxford Nanopore technologies present different sequencing error rates that might impact results interpretation ([29] (error rate per read is ~5% (R9.4 flow cells) so use of appropriate pipelines is critical to obtain high-accuracy consensus sequences). Despite the elevated error rates observed in Oxford Nanopore sequencing reads, highly accurate consensus-level sequence determination can be achieved, with single nucleotide variants (SNVs) detected at >99% sensitivity and >99% precision above a minimum of ~60-fold coverage depth, thereby ensuring the suitability for SARS-CoV-2 genome analysis as previously suggested [30]. In addition, the World Health Organization has recommended the use of this technology and the ARTIC protocol for the genomic surveillance of SARS-CoV-2 that several authors have implemented worldwide [31,32,33]. In this scenario, our results are validated, as more than 60-fold coverage depth was achieved, which supports the reliability of our conclusions.

In conclusion, herein, we report, for the first time, a case of reinfection and two cases of persistence from Colombia using whole-genome sequencing. In our case and in the absence of clinical data, we could not determine whether the reinfection could have worsened the clinical outcome of the patients as in previous studies [18]. Future studies should consider increasing the number of patients with comprehensive follow-up of clinical data and outcome. Nevertheless, these findings demonstrate the pivotal need to continue studying the disease dynamics of COVID-19 using genome tracking in light of our evidence of reinfection and persistence. This study also reinforces the paramount importance of genomic surveillance for understanding SARS-CoV-2 evolutionary and infection dynamics and the importance of this information in order to design effective preventive strategies in light of the vaccination programs that have started to take place across South America, particularly in Colombia.

## Data Availability

Data of the genomes can be retrieved from GISAID under the numbers EPI_ISL_1040921, EPI_ISL_1040922, EPI_ISL_1040923, EPI_ISL_1040924, EPI_ISL_1040925 and EPI_ISL_1040926.

## Supplementary Materials

The following are available online at https://www.mdpi.com/2076-393X/9/3/282/s1, Table S1: List of 4171 genomes used for the global comparison. Figure S1: Read depth variance found in the substitution sites of the six genomes sequenced herein: (A) Patient 1; (B) Patient 2; (C) Patient 3.
