# Supplementary figures and images for: Phylogenomic Evidence of Reinfection and Persistence of SARS-CoV-2: First Report from Colombia
Source: Vaccines (Basel). 2021 Mar 19;9(3):282. doi: 10.3390/vaccines9030282 (PMC8003345; doi:10.3390/vaccines9030282)

**A**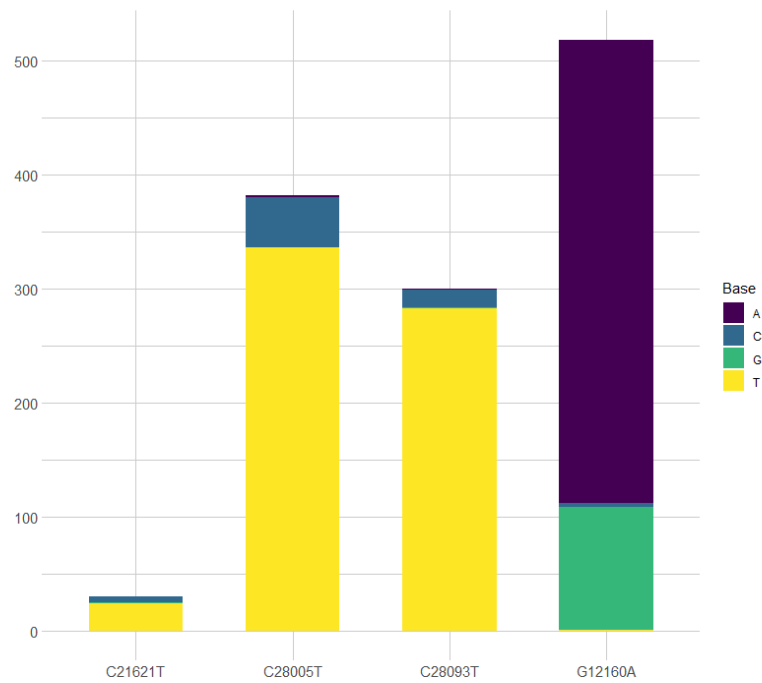**B**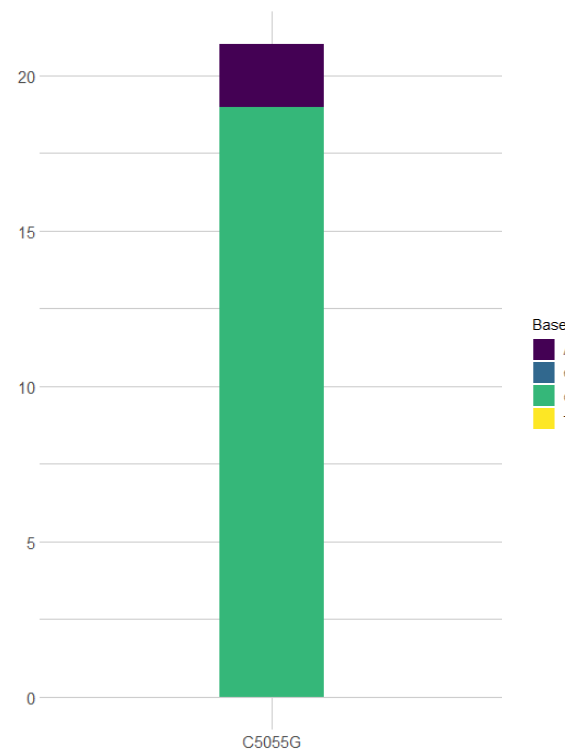**C**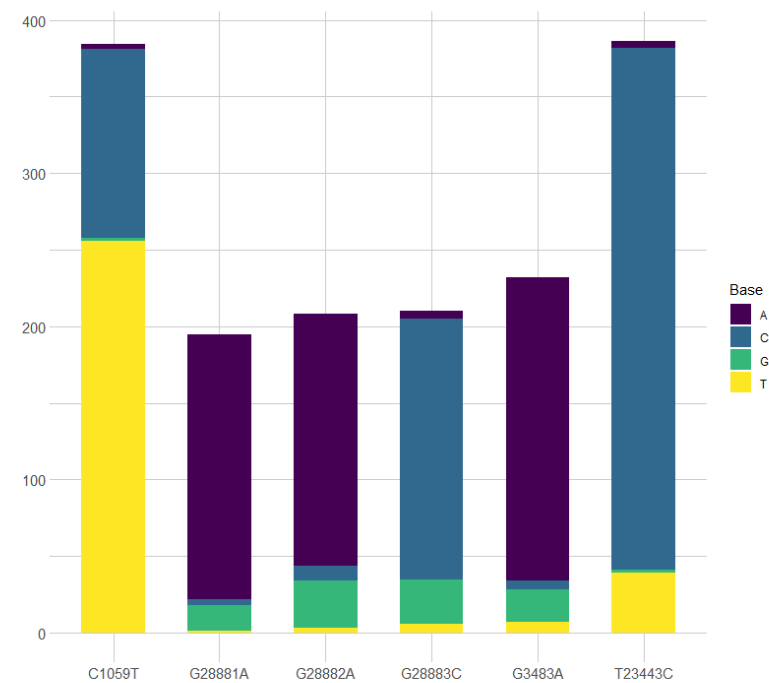

Supplement: Supplementary file 1 [file vaccines-09-00282-s001.zip › Supporting info/Supplementary Fig. 1 (3).pdf]
